# An analysis of the essential medicines policy in primary care: Findings from MedMinas project

**DOI:** 10.3389/fphar.2022.953329

**Published:** 2022-10-17

**Authors:** Tatiana Chama Borges Luz, Noemia Urruth Leão Tavares, Isabela Cristina Marques, Ana Karine Sarvel de Castro, Betania Barros Cota

**Affiliations:** ^1^ GETESA (Grupo de Estudos Transdisciplinares em Tecnologias em Saúde e Ambiente), René Rachou Institute (IRR), Oswaldo Cruz Foundation (FIOCRUZ), Belo Horizonte, Brazil; ^2^ Strathclyde Institute of Pharmacy and Biomedical Sciences (SIPBS), Strathclyde University, Glasgow, United Kingdom; ^3^ Department of Pharmacy, Faculty of Health Sciences, University of Brasília, Darcy Ribeiro University Campus, Brasília, Brazil

**Keywords:** essential medicines policy, medicines supply, mixed-methods study, primary health care, pharmaceutical services

## Abstract

**Background:** Essential Medicines Policy (EMP) has been adopted in Brazil to improve the provision and use of pharmaceuticals. This mixed methods study aims to bring evidence of the EMP implemented in municipalities in the context of primary care in Minas Gerais (20,997,560 inhabitants), Southeast Brazil.

**Methods:** We analysed the core output of the EMP, i.e., the municipal essential medicines lists (MEML) and the effects of the policy on the procurement and availability of medicines. Data sources included a sample of 1,019 individuals (patients, health managers and health professionals), 995 prescriptions, 2,365 dispensed medicines and policy documents from 26 municipalities. Data were collected between April and October 2019. Document analysis and thematic content analysis were performed, and four availability indexes were estimated.

**Results:** The findings suggest an overall lack of standardised and methodologically sound procedures to elaborate the MEML. Funding and public purchasing processes were found to be the major obstacles to medicine procurement. Only 63% of medicines were available at public community pharmacies and just 46.2% of patients had full access to their pharmaceutical treatment.

**Conclusion:** This study reveals weaknesses in the implementation of EMP and a clear disconnection between medicines selection, procurement, and availability, the three core elements of the supply system. These findings contribute to informing future policy improvement actions to strengthen this system. Other countries aiming to advance towards universal health coverage may learn from the challenges that primary care in Brazil still needs to address.

## Introduction

Brazil has a national healthcare system known as SUS—*Sistema Único de Saúde*, established more than 30 years ago, run by the government, funded through taxes, and covering the entire population of more than 210 million inhabitants (Paim et al., 2011; Brasil, 2021a).

The Brazilian’s National Medicines Policy (BNMP) was launched in 1998 (Brasil, 1998) as a fundamental component of SUS. Pharmaceutical Services based on Primary Care (PHCPS) are a programme, part of BNMP, involved in activities of regulation, planning, procurement, distribution, and dispensing of essential medicines in primary care related facilities, mostly public community pharmacies (PCPs) (Barreto and Guimarães, 2010; Brasil, 2013).

In accordance with the SUS principle of decentralization to subnational entities, Primary Care, and consequently the PHCPS, are coordinated by each of the 5,570 Brazilian municipalities, who are responsible for the development of local policies, health system management, and service provision (Facchini et al., 2018). Funding, on the other hand, is under shared responsibility of the federal, state, and municipal levels (Costa et al., 2017).

Despite significant incentives and guidance included in the Brazilian medicines-related legal framework throughout more than 20 years of the BNMP publication (Bermudez et al., 2018), there is still great difficulty in achieving the goals of the PCHPS in the country, especially regarding medicine supply and logistics. A nationwide study found an average availability of tracer medicines in PCHPS of only 52.9% (Nascimento et al., 2017), while a state-level study found an availability index verified by stock levels of 61.0% (Barbosa et al., 2021). To 9.7% of Municipal Health Secretaries the financial resources are perceived as sufficient to cover medicines demanded by patients (Faleiros et al., 2017). Additionally, PCHPS pharmacist managers reported lack of financial autonomy (61.5%), knowledge gaps on the financial resources available (81.7%), and lack of procedures for medicines selection, forecasting, and procurement (50%) (Gerlack et al., 2017). Considering that in Brazil universal access to medicines is constitutionally guaranteed to all citizens, litigation has been increasingly used by individuals to ensure their rights, which, in turn, affects health financing (Oliveira et al., 2020).

The PCHPS deem medicines to be the central element in guaranteeing comprehensive and continuous care for the population’s health needs and problems, both individually and collectively (OPS, 2013). Thus, two complementary systems must operate: 1) the supply system, encompassing medicines selection, procurement, and delivering; and 2) the pharmaceutical care system, including dispensing, counselling and monitoring, pharmacotherapeutic follow-up of patients and health education activities (Brasil, 2006a, 2009).

The Essential Medicines Policy (EMP) is embedded in the BNMP and isa cross-cutting dimension for the PHCPS management system in Brazil, as its main purpose is to promote free access to the population to efficacious, cost-effective, quality, and safe medicines (Bermudez et al., 2018). The main output following the EMP implementation is the Essential Medicines List (EML). The Brazilian National Essential medicines list—NEML—is known as RENAME (*Relação Nacional de Medicamentos Essenciais)*. The policy requires that states and municipalities develop their own lists based upon the NEML and conduct periodic reviews (Brasil, 1998). The pharmaceutical products selected in their lists must have adequate public sector financing and be continuously available to the healthcare system patients (Brasil, 1998, 2007; Bermudez et al., 2018; Brasil, 2021c).

Based on the premise that the EML constitute the benchmark for procurement processes and, consequently, for the availability of essential medicines in the healthcare system, this study brings evidence of EMP implemented in the municipalities. We examined the core output of EMP, i.e., the municipal lists of essential medicines—MEML– (*Relação Municipal de Medicamentos Essenciais,* REMUME). Also, we discussed the effects of the policy on its primary process and outcome, respectively: procurement effectiveness and barriers, and medicines availability at public community pharmacies (PCPs).

## Materials and methods

### Design and setting

This investigation is part of MedMinas Project (Luz, 2017), a mixed-methods study conducted in medium to large size-sized municipalities (35,000 to 900,000 inhabitants) from all 13 macro regions (Malachias et al., 2010) in the State of Minas Gerais (20,997,560 inhabitants) in the Southeast region of Brazil.

MedMinas adopted the principles of Rapid Evaluation Methods (REM) (Anker et al., 1993; McNall and Foster-Fishman, 2007), which recommends a sample of at least 20 health care facilities and 30 patients per facility. For the quantitative component, MedMinas used a multistage sampling technique in three levels of stratification: 1) macro-regions of the State of Minas Gerais; 2) municipalities within the macro-region; and 3) PHCPS facility. A sample size of 26 municipalities was estimated to guarantee the representativeness of the entire state. In each municipality, one service—a public community pharmacy was selected. Considering that 30 patients should be interviewed in each of the 26 services, a sample of 780 patients was selected. To this number a percentage of 20% was added to compensate for losses, totalling 936 patients of both sexes and aged 18 years or older and who were patients from the PCPs for at least 6 months.

For the qualitative component, a purposeful sample was estimated, totalling five key actors per municipality, divided in two subgroups: healthcare system managers, including the Municipal Health Secretary (1), the Director/Coordinator of Primary Health Care Services (1) and the Municipal Coordinator of Pharmaceutical Services (1); and health workers from the public community pharmacy, including a pharmacist (1) and a dispensary assistant (1).

### Data sources and data collection

Data were collected between April and October 2019, by a trained field team in each selected municipality. Data sources included in MedMinas comprised individuals (patients and professionals), prescriptions and dispensed medicines, and policy documents. Specific semi-structured, multidimensional, pre-tested, and piloted questionnaires were applied to each respondent profile. Face-to-face interviews were conducted at the public community pharmacies (PCPs) and at Municipal Health Secretariats (MHS). Patients were interviewed after dispensing, where information on their prescribed and dispensed medicines were also collected. Healthcare workers and managers were interviewed at their workplace. Policy documents were collected at the MHS. More details about MedMinas methods can be found in another publication (Luz et al., 2022).

### Data analysis

#### Document analysis of the Municipal Essential Medicines lists–MEML/REMUME

Copies of the current MEML were provided by the municipalities. A database was prepared combining the content of the lists. We classified all pharmaceutical products in accordance with the WHO-ATC/DDD system (WHO, 2021) and in accordance with the Brazilian Common Name (*Denominação Comum Brasileira*–DCB) that identifies the pharmaceutical substance or active pharmaceutical ingredient approved by the Brazilian Health Regulatory Agency–ANVISA (Brasil, 2021b). We also cross-referenced MEML with the Brazilian National Essential Medicines List (NEML/RENAME) (Brasil, 2018a).

In order to gain understanding and to be able to triangulate data (Bowen, 2009), we evaluated the MEML in two phases:

A) Appraisal of the overall content (WHO 2010; Rashid, 2016), estimating the following yes-no indicators: 1) presentation of the review committee members; 2) presentation of the criteria adopted for medicines’ inclusion/exclusion; 3) organization by level of care, indicating whether the proposed medicine should be listed for use (e.g., primary care, secondary care, tertiary care); 4) organization by dispensing facility (e.g., public community pharmacy, emergency care unit, psychosocial care centres, etc); 5) organization by therapeutic or pharmacologic classes (e.g., medicines listed by pharmacological or therapeutic groups); 6) organization by funding components (Basic, Strategic, and Specialized Component).

B) We included at this phase only medicines related to Primary Care. We used information provided by the MEML and, when the MEML did not specify, by the NEML. We excluded several products, such as those for hospital, specialized care and for internal use in health facilities. We also excluded items that are not considered medicines, such as food supplements and formulas, sunscreen, reagent strips, and lancets, among others. We estimated the indicators (mean/SD, minimum and maximum): 1) average number of medicines (i.e., pharmaceutical products defined by active ingredient/s, route of administration and strength); 2) average number of chemical substances. We aggregated medicines by therapeutic group and estimated the indicator “proportion of medicines listed in the MEML by anatomical main group (ATC 1st level).”

Additionally, we analysed managers and professionals’ responses to the following questions (yes/no answers), regarding primary care medicines, using descriptive statistics: *“In your opinion, the MEML is updated?”*; *“Do patients look for drugs that are not included in the MEML?”* We also analysed responses to the question *“In your opinion, is the MEML adequate to patients’ needs?”* (adequate/partially adequate/inadequate).

### Medicines procurement effectiveness and barriers

Concerns and experiences of managers and professionals may remain invisible if they are not properly acknowledged; hence, we investigated medicines procurement effectiveness and barriers, according to their perceptions. We analysed responses to the following questions (yes/no answers), using descriptive statistics: “*Has your municipality been able to procure primary care medicines*?“; “*Are there any difficulties purchasing these medicines*?” If the respondent stated “yes” to the latter, we asked an additional open-ended question: “*Could you explain why the municipality is not being able to purchase the medicines*?” Answers were analysed by Thematic Content Analysis (Bardin, 2011; Patton, 2015), which included codification of the answers in *units of meanings*. In sequence, similar codes were grouped to generate themes and categories. A coding frame was developed based on a Brazilian guideline and a technical note (Brasil 2006a; Brasil, 2014). Each final category was organized into larger themes following the coding frame and then that coding frame was applied to all data.

### Medicines availability at public community pharmacies

To estimate medicines availability at PCPs we considered four sources of information per municipality: patients’ data, prescriptions, dispensed medicines, and the MEML.

We asked patients if they had obtained the prescribed medicines and, if so, if they were given the amount needed for the duration of their treatment. We extracted from prescriptions and medicines dispensed the products’ names, dosage forms, and strengths. For analysis, we included only medicines prescribed and dispensed that were listed on the MEML. We classified the included products according to the WHO-ATC/DDD system (WHO, 2021).

We built four availability indexes (Bueno et al., 2021): 1) Overall medicines availability, 2) Medicines availability by therapeutic groups, 3) Prescription availability, and 4) Pharmaceutical treatment availability.

The analyses were conducted according to the following steps:

1) First, each prescribed medicine was classified “available” or “unavailable” to build the index “Overall medicines availability”. To be considered “available”, the product should be dispensed in the correct quantity for treatment duration. We estimated the index by using the formula:

$$\frac{Number\;of\;medicines\;available\;at\;the\;PCP}{Total\;number\;of\;prescribed\;medicines}$$

2) Then, medicines were aggregated by their main therapeutic groups and the proportions of prescribed and dispensed drugs were estimated (by ATC 1st level) to build the index “Medicines availability by therapeutic groups” by the formula:

$$\frac{Number\;of\;medicines\;available\;at\;the\;PCP\;by\;their\;main\;ATC\;group}{Total\;number\;of\;prescribed\;medicines\;by\;their\;main\;ATC\;group}$$

3) We built the index “Prescription availability”, considering each patients’ prescriptions. Each prescription was classified in one of the three following categories: “Totally filled” (if all prescribed medicines were considered available); “Partially filled” (if at least one prescribed medicine was considered unavailable) and “Unavailable” (if all prescribed items were unavailable). The index was estimated by the formula:

$$\frac{Number\;of\;prescriptions\;\left(totally\;filled/partially/unavailable\right)}{Total\;number\;of\;prescriptions}$$

4) Next, we built the index “Pharmaceutical treatment availability” considering all the prescriptions dispensed per patient (sometimes patients receive multiple prescriptions). Each treatment was classified in one of the three following categories: available (if all prescriptions were considered totally filled), partially available (if at least one prescription was considered partially filled) and unavailable (if all prescriptions were considered unavailable). The index was estimated by the formula:

$$\frac{Number\;of\;treatments\;\left(available/partially\;/unavailable\right)\;}{Total\;number\;of\;patients}$$

## Results

MedMinas included 26 municipalities varying from 37,784 to 409,341 inhabitants totalling 3,874,247 people. A total sample of 1,019 individuals participated in the study from three groups: managers (*n* = 77), health professionals (*n* = 50), and primary care patients (*n* = 892). The group of managers was represented by 24 municipal health secretaries, 27 coordinators of primary health care services and 26 coordinators of pharmaceutical services. One municipal health secretary refused to participate and another one asked to be replaced by the Municipal Director of Health Care. The group of health professionals consists in 26 dispensary assistants and 24 pharmacists, because in two municipalities, at the time of data collection, the coordinators of pharmaceutical services were also responsible for the public community pharmacies investigated, reducing the number of pharmacists in the study. Most study participants were women, with mean age ranging from 36.8 to 53.0 years, depending on the study group (Table 1).

**TABLE 1:** Sociodemographic profile of study participants. MedMinas Project, 2019.

Characteristic	n (% or SD)		
	Managers (*n* = 77)	Health professionals (*n* = 50)	Patients (*n* = 892)
Sex (female)	54 (70.1)	40 (80.0)	561 (62.9)
Age [mean (SD)]	42.7 (10.8)	36.8 (9.7)	53.0 (15.5)
Educational level (years of study)			
0 ≥ 9	-	-	582 (65.3)
10 to 13	1 (1.3)	16 (32.0)	236 (26.5)
≥14	76 (98.7)	34 (68.0)	73 (8.2)

### The municipal essential medicines lists

The EMP is implemented in the totality of municipalities included in the study (*n* = 26), but one municipality did not provide a copy of the MEML for our analysis. The evaluation of the content of the MEML showed that around a third presented the review committee members and only two lists provided the criteria adopted for medicines’ inclusion/exclusion. One quarter of the lists were organized by level of care, 48% were organized by dispensing facility, and 44% by therapeutic or pharmacologic classes. Three MEML were organized by funding components (Table 2).

**TABLE 2:** Findings from the Municipal Lists of Essential Medicines (REMUME) evaluation in accordance with the document analysis. MedMinas Project, 2019.

REMUME evaluation	n (%)
Document analysis	
Presentation of the review committee members (yes)	7 (28.0)
Presentation of the criteria adopted for medicines’ inclusion/exclusion (yes)	2 (8.0)
Frequency of REMUMEs organized by level of care^a^	7 (28.0)
Frequency of REMUMEs organized by dispensing facility^b^	12 (48.0)
Frequency of REMUME organized by therapeutic/pharmacologic classes	11 (44.0)
Frequency of REMUME organized by funding components^c^	3 (12.0)
Average number of medicines	
Mean (SD)	158.3 (38.0)
Minimum	95.0
Maximum	242.0
Average number of chemical substances	
Mean (SD)	105.6 (22.9)
Minimum	67
Maximum	159
Proportion of medicines by anatomical main group (ATC 1st level) (*n* = 3957)	
Nervous system (N)	954 (24.1)
Cardiovascular system (C)	661 (16.7)
Anti-infectives for systemic use (J)	530 (13.4)
Alimentary tract and metabolism (A)	419 (10.6)
Respiratory system (R)	266 (6.7)
Genito urinary system and sex hormones (G)	196 (5.0)
Systemic hormonal preparations (H)	194 (4.9)
Blood and blood forming organs (B)	168 (4.2)
Antiparasitic products, insecticides, and repellents (P)	162 (4.1)
Musculo-skeletal system (M)	142 (3.6)
Other^d^	265 (6.7)

^a^ Level of care: primary care, secondary care, tertiary care.

^b^ Dispensing facility: public community pharmacy, emergency care unit, psychosocial care centres, etc.

^c^ Funding components: basic, strategic, and specialized component.

^d^ Other: Dermatologicals (D), Sensory Organs (S) Various (V), Antineoplastic and Immunomodulating Agents (L), herbal medicines.

The total number of pharmaceutical products listed for primary care, considering all the MEML, was 3957 (mean 158.3 [SD ± 38.0]), while the total number of chemical substances was 2641 (mean 105.6 [SD ± 22.9]). Medicines from the nervous system (N), cardiovascular system (C), anti-infectives for systemic use (J), and alimentary tract and metabolism (A) were the most frequently included in the lists (64.8%) (Table 2).

Considering professionals’ perceptions regarding the MEML, the lists were updated for 64.9% of the managers and 82.6% of health professionals. However, 95.9% of health managers and 100% of health professionals declared that there is a demand from patients for medicines not covered by their lists. Regarding the adequacy of the lists in relation to patients’ needs, most managers (70.3%) and health professionals (82.6%) considered their lists adequate (Table 3).

**TABLE 3:** Managers and health professionals’ perceptions regarding the Municipal Lists of Essential Medicines (REMUME). MedMinas Project, 2019.

REMUME evaluation	n (%)
Perceptions of managers and health professionals	
Updated medicines list (yes)	
Managers (*n* = 77)	48 (64.9)
Health professionals (*n* = 23)^a^	19 (82.6)
Demands for medicines not covered by the list	
Managers (*n* = 77)	70 (95.9)
Health professionals (*n* = 50)	48 (100.0)
Adequacy to patients’ needs (yes)	
Managers (*n* = 77)	52 (70.3)
Health Professionals (*n* = 23)^a^	19 (82.6)

^a^ Questions not applied to dispensary assistants since these are out of the scope of their responsibilities at PCPs.

### Medicines procurement effectiveness and barriers

Regarding medicines procurement effectiveness, most managers and a portion of health professionals stated that their municipality has been able to procure primary care medicines (63.2% and 40.0% of respondents, respectively). Coincidently, a similar proportion of managers recognized difficulties to purchase medicines (62.3%) and 47 of them reflected on these difficulties. The major themes regarding procurement barriers yielded during thematic analysis and frequency of appearance are presented in Table 4. Funding (61.7%) and purchasing processes at SUS (38.3%) appeared in most of the responses.

**TABLE 4:** Managers and health professionals’ perceptions regarding medicines procurement. MedMinas Project, 2019.

Procurement	n (%)	
	Managers (*n* = 77)	Health professionals (*n* = 50)
Municipality being able to procure medicines (yes)	48 (63.2)	20 (40.0)
Difficulties to purchase medicines^a^ (yes)	48 (62.3)	-
Major themes regarding difficulties to purchase medicines^a^ ^,^ ^b^		
Funding	29 (61.7)	-
Purchasing processes at SUS	18 (38.3)	-
Pharmaceutical market	7 (14.9)	-
Litigation	2 (4.3)	-
Governance	1 (2.1)	-

^a^ Questions not applied to health professionals since these are out of the scope of their responsibilities at PCPs.

^b^ Response percentages exceed 100% because questions allowed respondents to mention multiple themes.

### Theme 1: Funding

Two subthemes emerged from this category: transfer of resources allocated to PHCPS and overall programme funding. In Brazil, federal, state and municipal government share the responsibility for funding the PHCPS programme. The resources allocated to PHCPS is transferred on a regular basis to the Municipal Health Fund. Most managers (18/47) were consistently concerned about the transfer of resources allocated to PHCPS by the State Government. They also complained about the poor financial situation of the municipalities (12/47).

“*Most of all, is the financial situation of the municipality… we have the State of Minas Gerais with an extremely complicated situation, from the financial point of view, in terms of transferring resources to the municipalities, especially in matters of healthcare. So, the municipality ends up paying for many things that were, previously, under the responsibility of the state. We have been suffering a lot from this!* (*Municipal Health Secretary, 24*).

“*What makes it difficult [medicines procurement] is the debt of the government of State of Minas Gerais with us. They are not transferring the funds. (Municipal Health Secretary, 16).”*

“*The problem that we’re going through is due to the lack of financial resources. We purchase medicines, but there are delays in the payment, and then the suppliers suspend the deliveries* *…*” *(Municipal Coordinator of PHCPS, 11)*

“*Financial resources! Our biggest challenge here is financial resources, we don´t have any money!” (Municipal Coordinator of PHCPS, 22)*

The underfunded situation of the PHCPS programme, however, was stressed by some managers.

“*The financial resources for the PHCPS programme are not enough. We are not being able to purchase several anti-hypertensives, antidiabetics, antidepressants…” (Municipal Coordinator of PHC Services, 27)*

“*I’m talking about the amount of money that comes, I mean, I’m considering the amount of money available to fund the programme. We have been working with the given budget, of course, but if we could increase the funding from the three levels of government, right, it would be great! We could expand our capacity of supply and everything…” (Municipal Health Secretary, 2)*

### Theme 2: Purchasing Processes at Sistema Único de Saúde (SUS)

Several constrains and challenges still exist and affect the capacity of the purchasing process of medicines by SUS. Managers (19/47) complained about difficulties in preparing public procurement tender documents, excessive bureaucracy, significant delay in public procurement tenders and bidding processes, and about the fact that many suppliers decide not to respond to public procurement tenders or not deliver the medicines at the end of the processes.

“*Well, the Statement of Requirements [the document that defines the product or service, that is, being put to tender] preparation is exhausting! We write it every single year and every year there is something to be changed! So, this document goes to City Hall because we need the feedback of the legal team and it keeps coming back and forth several times…” (Municipal Coordinator of PHCPS, 8)*

*“… bidding here is extremely slow, there are so many bids for the municipality to handle, so the processes are quite slow. I’ve participated in the preparation of a public bidding months ago, and so far, it has not been published yet.”* (*Municipal Coordinator of PHCPS*, 24)

“*The main difficulty is to involve the suppliers; we often cannot find them, biddings fail… we need the medicines, but they do not offer them for us to buy, there just are no suppliers!” (Municipal Coordinator of PHCPS, 15)*

One manager stated that the Brazilian legal rules on public procurement bidding (Brasil, 1993) need to be updated.

*“Umm, well, the great difficulty is the law itself, right, law 8666/93 [number of the Law], which is a total obstacle in the country, not just for purchasing medicines. We need something different, something newer, less bureaucratic, because the more bureaucratic the easier the deviations and errors, right?* (*Municipal Coordinator of PHCPS*, 6)

Other managers complained about the policy *Pharmaceutical Care Regionalization Strategy* (ERAF) published by the State Health Secretariat, that pre-select the suppliers for a comprehensive list of primary care medicines. The municipalities can, then, purchase direct from these suppliers. However, some managers believe that this policy adversely affected the effectiveness of the procurement processes.

“*We adhered to the ERAF policy, but the scarcity of the financial resources from the State is making us purchase small quantities of medicines. Maintaining such a low stock is leading us to re-do purchases within a very short time. Additionally, due to the lack of items in the State list, we run out of medicines we need.” (Municipal Health Secretary, 22)*

*…*“*Some suppliers were not willing to adhere to the State pharmaceutical pricing when selling the medicines to us*…*we had to re-specify ceiling prices to be able to purchase primary care medicines” (Municipal Health Secretary, 21)*

### Theme 3: Pharmaceutical market

Medicines procurement is a complex process and negotiations highly depend on market constraints. Managers (7/47) emphasized obstacles such as unreliable suppliers or lack of competitiveness.

“*We are having problems with the suppliers that win the bids because some of them do not have the medicines that were solicited in the public bidding, so we do not receive these medicines. Additionally, they are dividing deliveries over multiple delivery points, without our consent…so, you do purchase, you do plan, you do think about your deadline and at the end of the day there is no agreement between the supplier and you…” (Municipal Coordinator of PHCPS, 7)*

“*The problem of having just one supplier sometimes is they leave us in the lurch!” (Municipal Health Secretary, 17)*

“*They win the bid but don´t want to deliver the medicines they sold; they ask us to change the brand of the products.” (Municipal Coordinator of PHC Services, 12)*

“*…* *when we discuss prices, the suppliers want to sell medicines above the established ceiling prices and we don’t understand the reasons for that.” (Municipal Coordinator of PHCPS, 15)*

### Themes 4 and 5: Litigation and governance

A few respondents mentioned the themes litigation and governance as barriers to medicines procurement.

“*The main problem today is the judicialization of healthcare. We must buy medicines to attend the judiciary determination. But the lawsuits come from one person or from a small group of persons and this causes a lot of problems for our administration. We are not serving an entire population. We need to purchase small quantities of medicines at higher prices.” (Municipal Coordinator of PHCPS, 26)*.

One manager reflected about lack of governance when it comes to the procurement of medicines.

“*We have internal challenges, you know…The Municipal Health Fund today is under the control of the Secretariat of Finance, so we must get their budgetary approval, otherwise we cannot purchase medicines. We had to pick a fight with them…” (Municipal Coordinator of PHC Services, 27)*

### Medicines availability at public community pharmacies

**FIGURE 1:**
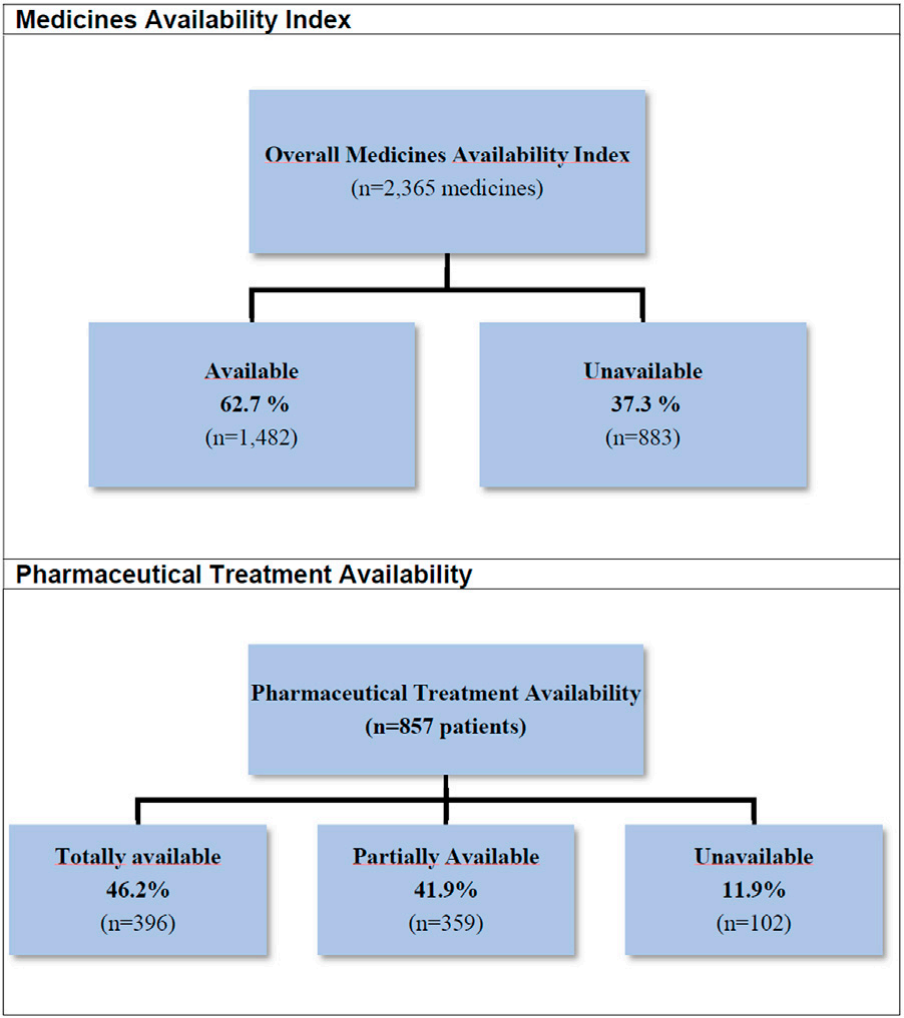
Medicines and pharmaceutical treatment availability at public community pharmacies. MedMinas Project, 2019.

We analysed a total of 995 prescriptions from 857 patients (mean 1.1 prescription per patient). From the total of 2,753 prescribed medicines, 2,365 (85.9%) were listed at MEML and were included in the analysis. The availability of medicines was 62.7%, i.e., medicines that were dispensed to patients in the right quantities for treatment duration. Only 396 patients (46.2%) had full availability to their pharmaceutical treatment (Figures 1A,B).

**FIGURE 2:**
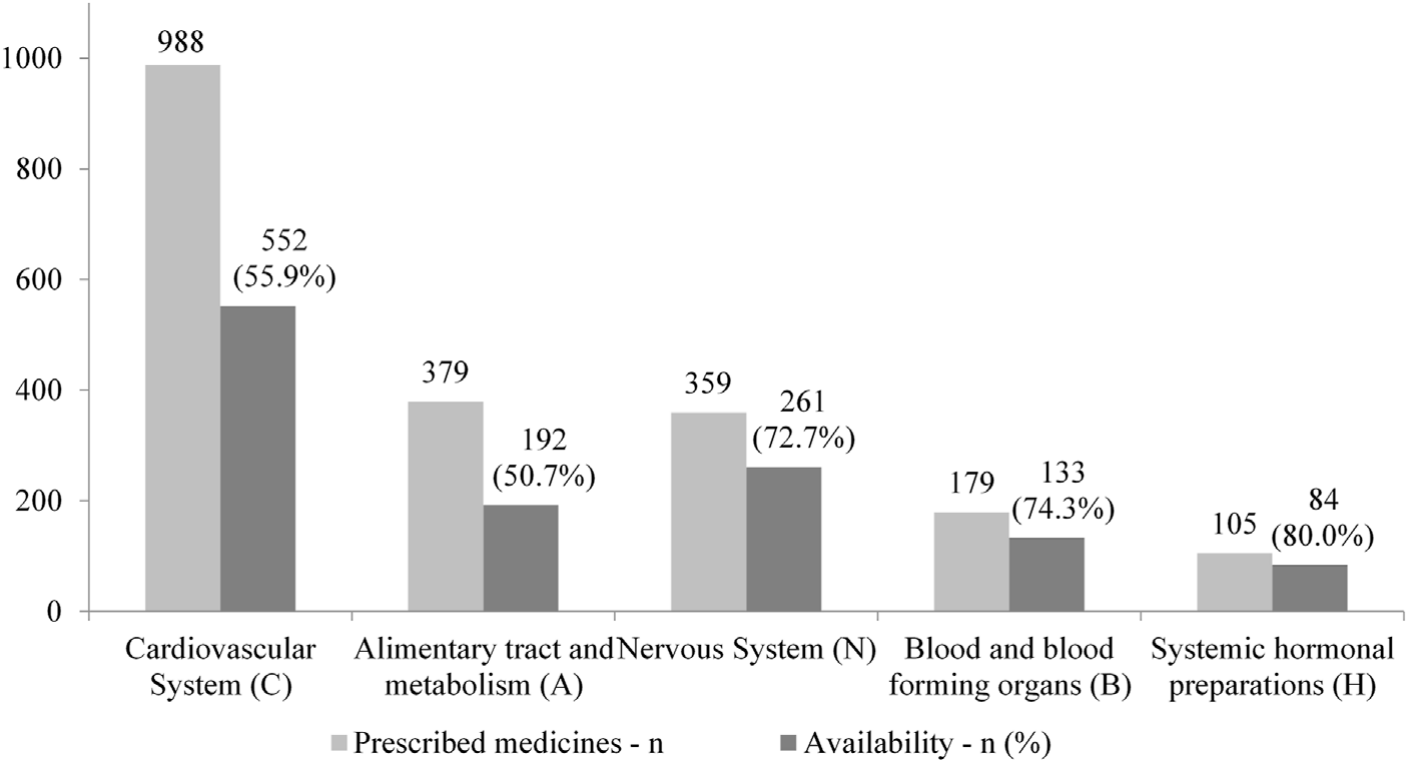
Top five prescribed therapeutic group (ATC 1st level) and availability at public community pharmacies. MedMinas, 2019.

Five main therapeutic groups (ATC 1st level) corresponded to 85% of the prescriptions: cardiovascular medicines (41.8%), alimentary tract and metabolism (16.0%), nervous system (15.2%), blood and blood forming organs (7.6%), and hormonal preparations (4.4%). Figure 2 displays these groups in accordance with the correspondent availability at public community pharmacies (PCPs). Alimentary tract and metabolism and cardiovascular medicines were the less available (50.7% and 55.9%, respectively) while hormonal preparations showed the highest availability (80.0%) at PCPs.

## Discussion

EMP is considered the core of the global health and development agenda (Wirtz et al., 2017), yet the availability of essential medicines is still substandard worldwide (Bazargani et al., 2014).

For almost 23 years, Brazil has been adopting EMP to improve provision and stimulate the rational use of pharmaceuticals in the country (Osorio-de-Castro et al., 2018). Despite of the cumulative experience and tradition of adopting EMP in Brazil for decades, little is known about the main EMP output—the essential medicines list—and the impact on procurement processes and availability of medicines. This study bridges this gap, providing evidence from primary care at the largest Brazilian state in number of municipalities and the second most populous (20,997,560), Minas Gerais (Brasil, 2021b).

The Brazilian National Essential Medicines List (NEML/RENAME) is the main guideline for municipalities planning their own lists. However, the document analysis showed lack of standardisation of the municipal lists (MEML/REMUME) regarding its presentation and formats, especially in the description of medicines and chemical substances, and in respect to relevant information about the pharmacological properties of the drugs or the systems the drugs act on. Only part of the MEML offered information referencing the level of care or dispensing facility for each listed medicine and very few provided the criteria adopted for medicines’ inclusion/exclusion and presented the committee members responsible for updating the list.

We also found several differences between MEML and the NEML concerning the overall organization of the lists. Particularly, we found a significative disparity in the number of chemical substances and medicines related to primary care. At the time of data collection, the NEML had 179 chemical substances and 364 medicines (Brasil, 2018a), numbers that were 1.7 and 2.3, respectively, higher than the average numbers for the municipalities.

Little attention is given to document analysis of MEML to allow direct comparisons, but investigations conducted in the South of Brazil showed similar findings. In respect to the average number of medicines presented, for instance, while we found a mean number of 158.3, Salvi et al. (2018) and Assunção et al. (2013) reported average numbers of 160.3 and 155.5, respectively. Relating to the distribution of the listed medicines by therapeutic groups, they also noticed a predominance of the nervous and cardiovascular systems on the MEML.

Taken together, our findings suggest an overall lack of adoption of standardised and methodologically sound procedures to elaborate the MEML (Rashid, 2016; Wright, 2017). Additionally, it seems that the NEML is not guiding these procedures either, given the contrast regarding the content of the national list in comparison with the municipal lists (Brasil, 2018a).

Managers and health professional perceptions regarding MEML, however, tell a different history. Even recognizing the existence of demands for medicines not covered by their lists, most of the professionals consider the lists updated and adequate to patients’ needs. These results differ from a nationwide investigation (Karnikowski et al., 2017) that showed a much higher percentage of managers perceiving the list as updated (80.4%), a much lower percentage of health workers perceiving demands for medicines not covered by the list (66.5% of the physicians) and considering the list adequate do patients’ needs (70.9% of the professionals responsible for the dispensing of medicines). While Karnikowski et al. (2017) included professionals responsible for PHCPS, professionals responsible for dispensing of medicines and physicians, we included three levels of municipal healthcare system managers and two groups of healthcare workers from PCPs (pharmacists and dispensary assistants). In contrast to the above-mentioned study, we proposed the questionnaires to all of them; thus, our results reflect a combination of a more varied set of perceptions.

One of the expected impacts of the implementation of the EMP was the contribution to a more efficient and regular medicines supply system in the municipalities. Within this context, the MEML should guide medicines procurement, supporting the decision-making process (MSH, 2012). Our findings, considering procurement processes and medicines availability at PCPs, do not confirm this assumption.

Most managers agreed on the existence of difficulties regarding medicines procurement, highlighting a range of barriers related to funding, purchasing processes by SUS, the pharmaceutical market, litigation, and governance. These are expected results considering that, despite the fact that the main focus of the BNMP is supply and logistics (Brasil, 1998), medicines provision in Brazil has always been an issue of concern (Brasil, 2018b).

Particularly, funding of the PHCPS programme and purchasing processes at SUS were considered the central obstacles for medicine procurement according to managers. In relation to funding, managers complained about the inadequate coverage of financial resources for the PHCPS programme and about irregularities in budget transferring. In Brazil, the PHCPS programme is co-funded by federal, state, and municipal governments. Federal and state governments conduct funding transfers to municipalities and municipalities are responsible for programme execution (Vieira, 2010; Brasil, 2017). The programme’s functioning depends on intergovernmental negotiation, especially regarding budget decisions (Costa et al., 2017). Our findings are in line with previous investigations (Faleiros et al., 2017; Mattos et al., 2019). A nationwide study showed that only 9.7% of municipal health secretaries considered the programme resources sufficient to meet the demands of the population (Faleiros et al., 2017) and an in-depth study showed that managers unanimously agreed that the PHCPS programme is underfunded. In respect to states’ transfers to their municipalities, other authors also pointed out the same issues (Brasil 2017; Mattos et al., 2019).

In MedMinas, managers noticed shortcomings related to municipal financial resources to fund the PHCPS programme. One previous investigation evaluated the allocation of financial resources in medicines procurement of 960 Brazilian municipalities, showing that 73% applied a financial value below that recommended by the legislation (Pontes et al., 2017; Tavares et al., 2017). It is possible that the precarious financial situation that emerged from our data is correlated to those results.

Of relevance were the findings related to the capacity of public buyers to execute efficient purchases: ex-ante, when preparing tender documents; during tender processes; and, ex-post, while managing contracts. Managers perceived the process as excessively bureaucratic, time-consuming, and dependent on unreliable suppliers. Information about barriers related to medicines procurement in Brazil, especially for primary care, is scarce, but our results are consistent with the available evidence (Brasil, 2006b; Mattos et al., 2019). It is worth mentioning the managers’ complaints concerning abusive sale prices, even after the implementation in Minas Gerais of the policy ‘*Strategy of Pharmaceutical Services Regionalization*—ERAF’, conceived to improve medicines procurement and distribution within the state (Minas Gerais, 2016), also consistent with the literature. Pontes et al. (2017) showed that, of the 20 most purchased medicines, 19 had an average unit price above the reference price.

The availability of medicines at PCPs is one of the ultimate goals of the implementation of EMP. According to WHO, essential medicines should be continuously available within healthcare systems, in adequate amounts, in the appropriate doses, with assured quality (Laing et al., 2003). We evaluated almost 1,000 prescriptions and more than 2,300 prescribed medicines at primary care and found substandard levels of availability. Dispensing of 100% of the medicines prescribed to patients is the ideal value (Teni et al., 2022), but only 63% of medicines were available. Additionally, the full prescribed treatment was dispensed to just 46.2% of patients, rates lower than a previous study (Nascimento et al., 2017), but similar to another study in primary care we recently published (Bueno et al., 2021).

We analysed medicines availability using a combination of individual-level data sources. Therefore, differently from other assessment strategies (e.g, WHO, 2008; Rocha et al., 2021), our method allowed us to better evaluate the demand for medicines, comparing prescribed versus actually dispensed medicines at primary care. Cardiovascular medicines and drugs acting on the alimentary tract and metabolism were, at the same time, the most prescribed (41.8% and 16.0%, each) and the least available therapeutic groups (55.9% and 50.7%). Of the medicines analysed, 85.9% were listed in the MEML and the prescription pattern we found is largely coincident with our previous findings (Bueno et al., 2021). This evidence suggests a good level of prescriber adherence to the MEML and it is possible that prescribers are not being fully adherent to the lists because they mistrust the supply system. There are, however, considerable differences between the MEML’s overall profile and the most prescribed therapeutic groups. In MEML, medicines for the nervous system clearly predominate (24.1%), with cardiovascular drugs appearing in second (16.7%) and alimentary tract and metabolism only in fourth (10.6%). Even though no extensive conclusions can be drawn, apparently the MEML are not strictly reflecting the clinical needs of primary care patients. If this is the case, it is possible that both over and understocking are occurring at PCPs and further studies are needed to better understand this point.

### Limitations

This study provided valuable insights into EMP implementation and impact; however, some limitations must be acknowledged. We used interviews to collect part of the data, which is subject to selection and information biases. We managed such interferences by using pre-tested and piloted instruments and by employing trained interviewers that followed standardized procedures during fieldwork. Patients were interviewed after dispensing, with the presentation of medical prescriptions and medicines, minimizing the risk of memory bias (Luz et al., 2022). We adopted the principles of Rapid Evaluation Methods, so our patient sample cannot be considered representative of the Brazilian primary care population. However, the participants’ characteristics are similar to those studies that included large samples of patients (Guibu et al., 2017; Bueno, Simões and Luz, 2021). MedMinas is a mixed-methods study that does not rely on a single research paradigm, thus allowing the extrapolation of the results to other Brazilian municipalities, especially medium to large-sized cities.

### Implications for policy and practice

Medicines selection, procurement, and availability are core elements of the supply system in which each of these elements builds on the previous and leads to the next, cyclically (MSH, 2012). The Essential Medicines Policy (EMP) at PHCPS in Brazil follows the same rationale, i.e., its central purpose is to select evidence-based, quality, and cost-effective medicines in the adequate formulation and dose. These products need to be offered in the amounts that satisfy the population’s health needs and must be rationally prescribed, dispensed at PCPs, and correctly used by patients (Vasconcelos et al., 2017; Bermudez et al., 2018).

Theoretically, one of the major benefits of implementing an EMP is achieving more regular and efficient medicines supply processes (Bazargani et al., 2014); however, our evidence does not support this assumption. Overall, our results point to weaknesses in the EMP implementation. Several key elements are missing, such as stakeholder engagement, individual accountability for the policy implementation and outcomes, assurance of funding provision, and monitoring and evaluation procedures (Wright, 2017). It would be recommended to apply change strategies to stimulate participation and planning for effective EMP implementation.

The EML, beyond than being seen as normative instruments, need to be understood as a guideline to ensure the rational use of medicines (WHO, 2012). The lists are meant to be incorporated into health service practices as sources of reliable information and guidance for several processes and activities. Ultimately, if these documents are viewed by managers and healthcare professionals as mere lists of medicines, their effectiveness for the healthcare system is compromised as they will not contribute to medicine coverage to the population. Therefore, it is necessary to develop a plan to influence the general culture toward the importance and use of the EML.

The evident disconnection between medicines selection, procurement, and availability also suggests that the pharmaceutical management framework is not guiding the supply system. The consequence of the breakdown we have identified is the failure of the entire process, thus harming healthcare patients. Investments should be made to ensure adequate funding and pharmaceutical planning and management.

## Conclusion

We have highlighted barriers to EMP implementation in primary care at SUS in Brazil, one of the largest public healthcare systems in the world. These findings contribute to informing future policy improvement actions to strengthen the supply system. Other countries adopting EMP and aiming to advance towards universal health coverage may learn from the challenges that Brazil still needs to address.

## Data Availability

The data that supported this study are not public, but may be shared upon reasonable request to the corresponding author.

## Abbreviations

BNMP: Brazilian’s National Medicines Policy; MEML/REMUME, Municipal lists of Essential Medicines (Relação Municipal de Medicamentos Essenciais, in Portuguese); EML, Essential Medicines List; EMP, Essential Medicines Policy; ERAF, Pharmaceutical Care Regionalization Strategy; MHS, Municipal Health Secretariats; NEML/RENAME, Brazilian National Essential Medicines List (Relação Nacional de Medicamentos Essenciais, in Portuguese); PCPs, Public community pharmacies; PHCPS, Pharmaceutical Services based on Primary Care; REM, Rapid Evaluation Methods; SUS, Brazilian National healthcare system (Sistema Único de Saúde, in Portuguese).
